# Role of Macrophage Phagocytosis as Predictive Marker of the Prevalence of Coronary Heart Disease and Acute Coronary Syndromes

**DOI:** 10.1155/cdr/9927583

**Published:** 2025-11-26

**Authors:** Yuming Wang, Wenjing Zhou, Qinyi Zhou, Changqing Du, Yimin Tang, Kefu Zhu

**Affiliations:** ^1^Department of Geriatrics, The First Affiliated Hospital, School of Medicine, Zhejiang University, Hangzhou, Zhejiang Province, China; ^2^Zhejiang Key Laboratory for Diagnosis and Treatment of Physic-Chemical and Aging-Related Injuries, Hangzhou, Zhejiang Province, China; ^3^LinkDoc RWE Research Institute, LinkDoc Technology, Beijing, China; ^4^Department of Cardiology, Zhejiang Hospital, Hangzhou, Zhejiang Province, China

**Keywords:** acute coronary syndromes, atherosclerotic cardiovascular disease, coronary heart disease, monocyte-derived macrophages, phagocytosis

## Abstract

**Background:**

Atherosclerotic cardiovascular disease (ASCVD) pathogenesis is closely associated with macrophages. This study sought to explore the role of phagocytosis by monocyte-derived macrophages (MDMs) in the blood in the context of coronary heart disease (CHD) and acute coronary syndromes (ACSs).

**Methods:**

This study employed a matched case–control design. Individuals with suspected CHD were recruited and allocated to a control cohort or a CHD cohort, with the latter further stratified into stable angina pectoris and ACS subgroups according to clinical diagnoses. Clinical data were collected, MDMs were isolated, and macrophage phagocytic activity was evaluated using fluorescent-labeled latex microspheres.

**Results:**

Macrophage phagocytic rates were significantly reduced in the CHD group relative to the control group, with further decreases observed in the ACS subgroup. Multivariable linear regression revealed that age, low-density lipoprotein cholesterol (LDL-C), high-sensitivity C-reactive protein (hs-CRP), and fibrinogen were independently and negatively correlated with macrophage phagocytic rates. Multivariable analyses suggested that diminished macrophage phagocytic rates were linked to an elevated risk of both CHD and ACS. Receiver operating characteristic (ROC) curve analysis identified the optimal cutoff values of macrophage phagocytic rates for predicting CHD and ACS as 62.6% and 63.4%, respectively, with the area under the curves (AUCs) measured at 0.679 and 0.669.

**Conclusions:**

Macrophage phagocytic activity is reduced in CHD patients, particularly in those with ACS. Diminished macrophage phagocytic function is linked to CHD and ACS. Macrophage phagocytosis could act as a protective biomarker in CHD and ACS, providing new insights into the pathophysiology of ASCVD.

## 1. Introduction

Coronary heart disease (CHD) stands as the predominant manifestation of atherosclerotic cardiovascular disease (ASCVD) and continues to be the leading cause of mortality globally [1]. Atherosclerosis (AS), the underlying pathological mechanism of CHD, is characterized by a multifaceted inflammatory response. Within atherosclerotic lesions, macrophages, originating from myeloid progenitors, constitute the principal immune cell population and are instrumental throughout the various stages of AS, encompassing lesion formation, necrotic core development, and plaque resolution. An early critical event in the development of AS is the migration of monocytes across the endothelial barrier into the arterial intima, a process facilitated by endothelial adhesion molecules and chemokine signaling pathways [2].

When the endothelium becomes activated, circulating monocytes traverse the endothelial barrier into the arterial intima, subsequently differentiating into macrophages within the developing lesion [3]. High levels of low-density lipoprotein cholesterol (LDL-C) and the subendothelial buildup of modified-oxidized LDL (ox-LDL) promote the recruitment and retention of monocytes and lymphocytes within the arterial wall. The accumulation of macrophages in the vascular intima is a defining feature of AS, where these macrophages ingest lipoproteins and transform into lipid-laden foam cells. In turn, foam cells release various inflammatory mediators, amplifying the inflammatory response and contributing to the progression of atherosclerotic lesions. The rupture of vulnerable coronary plaques underlies the pathophysiology of acute coronary syndromes (ACSs) [4].

Macrophage dysfunction represents a cardinal mechanism driving disease pathogenesis. Nevertheless, the role of macrophage phagocytic activity in both the circulatory system and arterial wall microenvironment remains incompletely understood. Thus, the present study is aimed at exploring the association between phagocytosis of monocyte-derived macrophages (MDMs) in blood and the presence and CHD and ACS.

## 2. Methods

### 2.1. Study Population and Definition

In this matched case–control study, participants with suspected CHD were enrolled in 2025 from the Department of Cardiology at Zhejiang Hospital, Hangzhou, China. The diagnosis and categorization of CHD, including stable angina pectoris (SAP) and ACSs, were performed following the guidelines of the American College of Cardiology and the American Heart Association [5]. This study was performed in strict compliance with the Declaration of Helsinki. The study protocol was approved by the Ethics Committee of Zhejiang Hospital (No. ZJHIRB-2025-090K).

The inclusion criteria stipulated that all subjects were aged over 18 years and had not used lipid-lowering medications or aspirin within the preceding month. The exclusion criteria were as follows: subjects with a 30%–50% coronary stenosis confirmed by coronary angiography (CAG); active infectious disease; hematologic disorder; thyroid disease; malignant tumor; severe hepatic, renal, or pulmonary insufficiency; or a history of transient ischemic attack, stroke, peripheral arterial disease, cardiomyopathy, congenital heart disease, valvular heart disease, or autoimmune disease. The control group comprised sex- and age-matched subjects without significant coronary artery disease, defined as CAG confirmed luminal stenosis < 30%. Diagnoses of hypertension and Type 2 diabetes mellitus (T2DM) in subjects were made according to the guidelines [6, 7] or current use of medications.

Subjects were categorized into a control group and a CHD group. Individuals in the CHD group were further stratified into SAP and ACS subgroups.

### 2.2. Clinical Data Collection

Comprehensive data collection encompassed demographic characteristics, medical history, clinical parameters, and biochemical indices. Specific information included age, sex, and body mass index (BMI); history of hypertension, T2DM, smoking, and alcohol consumption; blood counts (leukocytes, monocytes, lymphocytes, and neutrophils); biochemical markers (total protein, globulin, alanine aminotransferase [ALT], total bilirubin, triglyceride [TG], total cholesterol [TC], LDL-C, high-density lipoprotein cholesterol [HDL-C], fasting blood glucose [FBG], and glycated hemoglobin A1c [HbA1c]); renal function indicators (serum creatinine [SCr], urea, and uric acid); inflammatory markers (high-sensitivity C-reactive protein [hs-CRP] and fibrinogen); and imaging results (echocardiogram and CAG findings). All data collection procedures were subject to rigorous quality control measures to ensure accuracy and consistency.

### 2.3. CAG

CAG assessments included the left main coronary artery, right coronary artery (RCA), left anterior descending artery (LAD), and left circumflex artery (LCX). Lesions were considered significant if they exhibited ≥ 50% luminal narrowing. In this study, two experienced cardiologists independently reviewed the angiograms in a blinded manner using standard projections to ensure objectivity and reproducibility. When discrepancies arose between their evaluations, a third cardiologist was consulted to reach a consensus.

### 2.4. Peripheral Blood Mononuclear Cell (PBMC) Isolation, Differentiation, and Culture

Blood samples from patients with ACS were collected immediately upon admission into 3.0-mL EDTA-K_2_ anticoagulant vacuum tubes (Becton Dickinson, United States), whereas fasting blood samples from other subjects were collected the following morning. Residual peripheral blood samples from routine laboratory tests were obtained from enrolled subjects. PBMCs were isolated using Lymphoprep density gradient centrifugation (STEMCELL Technologies, Germany) according to the manufacturer's instructions. Monocytes were plated at a density of 1 × 10^6^ cells/mL in RPMI 1640 medium supplemented with 1% (*v*/*v*) penicillin–streptomycin (Gibco, United States). Cells were cultured at 37°C in a 5% CO_2_ incubator for 2 h in serum-free conditions to facilitate adhesion to tissue culture plates. Following adherence, the medium was replaced with RPMI 1640 containing 10% (*v*/*v*) fetal bovine serum (FBS, Gibco, United States) to promote macrophage differentiation.

Following a 24-h adherence period, isolated monocytes were cultured in RPMI 1640 medium supplemented with 10% (*v*/*v*) FBS, 1% (*v*/*v*) penicillin–streptomycin, and 20 ng/mL recombinant human macrophage colony-stimulating factor (rhM-CSF, BioLegend, United States). Cells were maintained at 37°C in a 5% CO_2_ incubator for 5 days to promote differentiation and proliferation into confluent MDMs [8]. Differentiated MDMs were maintained as unstimulated M0 macrophages until polarization. For M1 polarization, cells were treated with 50 ng/mL lipopolysaccharide (LPS, Sigma-Aldrich, United States) and 20 ng/mL interferon-*γ* (IFN*γ*) (Sino Biological, China) for an additional 24 h, maintaining culture conditions at 37°C with 5% CO_2_.

### 2.5. Macrophage Phagocytosis Assay Protocol

#### 2.5.1. Cell Seeding

Human M1-polarized MDMs were plated at a density of 5 × 10^5^ cells/mL in 24-well plates, with 0.5 mL of prewarmed (37°C) culture medium per well.

#### 2.5.2. Bead Incubation

Fluorescent latex beads (Sigma-Aldrich, United States) (4 *μ*L/mL) were added to the wells, and plates were incubated at 37°C in 5% CO_2_ for 2 h to allow phagocytosis.

#### 2.5.3. Cell Harvesting

Following incubation, nonadherent beads were removed by washing cells twice with prewarmed PBS. MDMs were gently detached by pipetting, and cell suspensions were collected for flow cytometry analysis.

#### 2.5.4. Flow Cytometry Analysis

The percentage of MDMs positive for latex bead phagocytosis was quantified using a CytoFLEX flow cytometer (Beckman Coulter, United States). Data were analyzed with FlowJo v10.8.1, gate-setting on viable cells and fluorescent bead-positive populations.

### 2.6. Statistical Analysis

Statistical analyses were performed using GraphPad Prism (Version 8, GraphPad Software Inc., United States) and IBM SPSS Statistics (Version 21, IBM Corp., United States). The Kolmogorov–Smirnov test was applied to assess the normality of data distributions. Continuous variables were presented as mean ± standard deviation (SD) for normally distributed data or as median with interquartile range (IQR, Q1–Q3) for nonnormally distributed data. Categorical variables were expressed as frequencies and percentages. For comparisons between two groups, independent samples *t*-tests were used for normally distributed data with equal variances, whereas the Mann–Whitney *U* test was applied for nonnormally distributed or heteroscedastic data. For comparisons among multiple groups, one-way analysis of variance (ANOVA) was conducted for normally distributed data with homogeneous variances, while the Kruskal–Wallis test was employed for nonnormally distributed or heteroscedastic datasets. When ANOVA indicated significant differences, the least significant difference (LSD) test was performed for pairwise comparisons. Differences in categorical variables were evaluated using the chi-square test or Fisher's exact test as appropriate. Linear relationships were analyzed using Pearson's correlation for normally distributed data and Spearman's rank correlation for nonnormally distributed data. To determine independent associations between predictor variables and macrophage phagocytic rates, linear regression models were constructed with adjustment for potential confounders. Logistic regression analysis was used to examine the independent relationship between macrophage phagocytic rates and the prevalence of CHD and ACS, controlling for confounders. The discriminatory ability of macrophage phagocytic rates for CHD and ACS was assessed using receiver operating characteristic (ROC) curve analysis, with area under the curve (AUC) values calculated to evaluate diagnostic performance. A *p* value < 0.05 was considered statistically significant.

## 3. Results

### 3.1. Baseline Clinical Parameters of Controls and CHD

A total of 270 CHD patients and age- and sex-matched controls and their baseline clinical parameters are presented in Table 1. The average age was 62.1 ± 11.0 years in the control group and 62.3 ± 12.0 years in the CHD group. Males comprised 66.7% (180/270) across both groups. Compared with controls, CHD patients showed significantly higher smoking prevalence and elevated levels of leukocytes, neutrophils, monocytes, TC, LDL-C, hs-CRP, and fibrinogen (all *p* < 0.05). By contrast, CHD patients had significantly lower levels of LVEF than controls (*p* < 0.01). No significant group differences were observed in demographic characteristics (age, sex, and BMI), comorbidities (hypertension and T2DM), CHD family history, drinking, medication use, systolic blood pressure (SBP), diastolic blood pressure (DBP), or laboratory parameters (lymphocytes, total protein, globulin, ALT, total bilirubin, TG, HDL-C, FPG, HbA1c, SCr, urea, and uric acid).

### 3.2. Baseline Clinical Parameters and CAG Findings in CHD Subgroups

CHD patients were stratified into SAP (*n* = 146) and ACS (*n* = 124) subgroups according to clinical diagnoses.  Table 2 presents the baseline clinical parameters and CAG findings of the two subgroups. Compared with the SAP group, the ACS group had significantly higher levels of leukocytes, neutrophils, monocytes, hs-CRP, and fibrinogen, as well as a lower age and less metformin use (all *p* < 0.05). CAG results showed that the ACS group had a significantly higher prevalence of triple vessel compared with the SAP group, primarily attributed to a greater number of lesions in the LAD and RCA (all *p* < 0.05, Table 2).

### 3.3. Macrophage Phagocytosis

**Figure 1:**
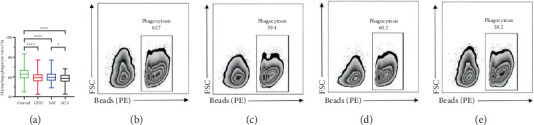
Flow cytometry analysis of (a) macrophage phagocytic rates and representative images of the (b) control (*n* = 270), (c) CHD (*n* = 270), (d) SAP (*n* = 146), and (e) ACS (*n* = 124) groups. A significant difference between groups is indicated at ⁣^∗^*p* < 0.05, ⁣^∗∗^*p* < 0.01, ⁣^∗∗∗^*p* < 0.001, and ⁣^∗∗∗∗^*p* < 0.0001. FSC, forward scatter.

Figure 1 depicts the intergroup comparison of macrophage phagocytic activity, as quantified by flow cytometry, between the control group, CHD group, and its subtypes. Macrophage phagocytic rates were 62.8% ± 5.4% in the control group (Figure 1b), 59.3% ± 5.8% in the CHD group (Figure 1c), 60.2% ± 5.9% in the SAP group (Figure 1d), and 58.2% ± 5.4%in the ACS group (Figure 1e). Compared with the control group, macrophage phagocytic rates were significantly lower in the CHD, SAP, and ACS groups (all *p* < 0.001, Figure 1a). Macrophage phagocytic rates in the ACS group were significantly lower than those in the SAP group (*p* < 0.05, Figure 1a).

### 3.4. Correlation of Macrophage Phagocytosis With Basic and Clinical Parameters

**Figure 2:**
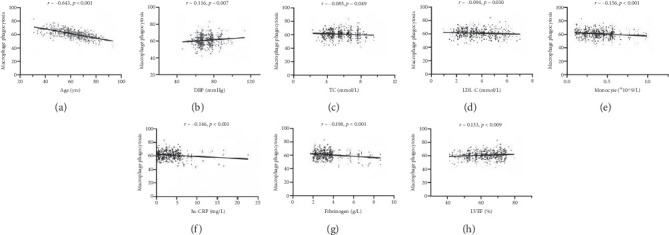
Correlation of macrophage phagocytosis with clinical parameters by Pearson correlation analysis (*n* = 540). (a) Age, (b) DBP, (c) TC, (d) LDL-C, (e) monocyte, (f) hs-CRP, (g) fibrinogen, and (h) LVEF.

The Pearson correlation analysis, conducted on macrophage phagocytic rates and basic and clinical parameters, showed a negative correlation between macrophage phagocytic rates and age (*r* = −0.643, *p* < 0.001, Figure 2a), TC (*r* = −0.085, *p* < 0.05, Figure 2c), LDL-C (*r* = −0.094, *p* < 0.05, Figure 2d), monocyte (*r* = −0.156, *p* < 0.001, Figure 2e), hs-CRP (*r* = −0.146, *p* < 0.001, Figure 2f), and fibrinogen (*r* = −0.198, *p* < 0.001, Figure 2g). A significant weak positive correlation was observed between macrophage phagocytic rates and DBP (*r* = 0.116, *p* < 0.01, Figure 2b) and LVEF (*r* = 0.133, *p* < 0.01, Figure 2h).

Multivariable linear regression analysis showed that macrophage phagocytic rates were negatively and independently associated with age (*β* = −0.355, *p* < 0.001), LDL-C (*β* = −0.853, *p* < 0.05), hs-CRP (*β* = −0.163, *p* < 0.05), and fibrinogen (*β* = −0.838, *p* < 0.001, Table 3).

### 3.5. Association of Macrophage Phagocytosis With CHD and ACS

Table 4 displays the results of univariate and multivariable logistic regression models evaluating macrophage phagocytic rates as predictors of CHD and ACS. Univariate analysis showed a significant negative association between macrophage phagocytic rates and the presence of CHD (OR = 0.891, 95% CI = 0.861–0.922, *p* < 0.001) or ACS (OR = 0.890, 95% CI = 0.857–0.925, *p* < 0.001). In multivariable models (adjusted for potential confounders), macrophage phagocytic rates were negatively and significantly associated with CHD (OR = 0.814, 95% CI = 0.776–0.854, *p* < 0.001 in Model 2; OR = 0.847, 95% CI = 0.802–0.894, *p* < 0.001 in Model 3) and ACS (OR = 0.748, 95% CI = 0.703–0.796, *p* < 0.001 in Model 2; OR = 0.747, 95% CI = 0.680–0.819, *p* < 0.05 in Model 3).

### 3.6. ROC Curve of Macrophage Phagocytic Rates in CHD and ACS

**Figure 3:**
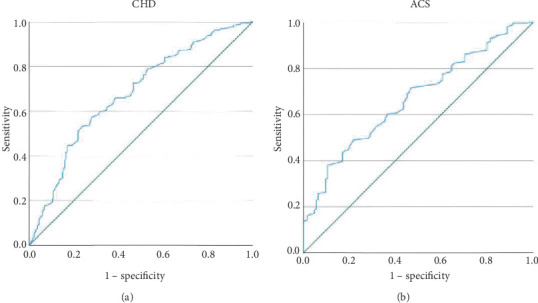
Receiver operating characteristic (ROC) curve of macrophage phagocytic rates in CHD and ACS: (a) CHD (AUC = 67.9%, *p* < 0.001); (b) ACS (AUC = 66.9%, *p* < 0.001).

ROC curve analysis demonstrated that macrophage phagocytic rates had significant predictive value for CHD and ACS, with AUC values of 0.679 (95% CI: 0.634–0.724, *p* < 0.001) and 0.669 (95% CI: 0.618–0.720, *p* < 0.001), respectively. The optimal cutoff values, determined by Youden's index, were 62.6% for CHD (sensitivity: 53.0%, specificity: 76.7%) and 63.4% for ACS (sensitivity: 38.2%, specificity: 89.5%; Figure 3).

## 4. Discussion

In this age- and sex-matched case–control study (*n* = 540), we systematically evaluated the independent association between macrophage phagocytic activity and the presence of CHD, with subgroup analyses distinguishing SAP and ACS.

The results revealed that macrophage phagocytic rates were significantly lower in the CHD group compared to the control group, with the most pronounced reduction observed in the ACS subgroup. Macrophage phagocytosis was independently related to an elevated risk of CHD and ACS. Additionally, correlation analysis showed that macrophage phagocytosis was strongly correlated with age and weakly correlated with TC, LDL-C, monocytes, hs-CRP, fibrinogen, DBP, and LVEF. These results indicate that macrophage phagocytosis might act as a protective marker in CHD, and the pathophysiology of ASCVD is intimately associated with macrophages. Further basic research is needed to elucidate the underlying mechanisms, and prospective randomized controlled trials are warranted to establish a causal relationship.

Monocytes and macrophages, key players in the innate immune system, originate from myeloid progenitor cells in the bone marrow and circulate in the bloodstream as precursor cells that can rapidly respond to inflammatory signals [9]. Circulating monocytes recognize pathogens and cellular debris through receptor-mediated pathways, serving as a link between the innate and adaptive immune systems. Once recruited into tissues, they differentiate into macrophages, which play essential roles in preserving local homeostasis, removing apoptotic cells, and regulating inflammatory responses. Disruption of these processes—such as impaired phagocytosis, defective efferocytosis, or excessive cytokine secretion—can result in chronic inflammation and contribute to the development of various diseases, including recurrent infections, sepsis, and AS [10].

AS is a chronic inflammatory condition that underpins CHD and is initiated by the retention of apolipoprotein B-containing lipoproteins within the arterial intima, leading to the recruitment of monocytes and their subsequent differentiation into macrophages [11]. MDMs may fail to effectively clear modified lipoproteins, transforming into proinflammatory foam cells. These foam cells release cytokines such as tumor necrosis factor-*α* (TNF-*α*) and interleukin-1*β* (IL-1*β*), exacerbating endothelial dysfunction and sustaining local inflammation [12]. Impaired efferocytosis within atherosclerotic plaques results in the buildup of apoptotic cells and necrotic material, contributing to the enlargement of a lipid-rich necrotic core. Vulnerable plaques—marked by a substantial lipid core, a thin fibrous cap, and a high density of macrophages—are susceptible to rupture, which exposes thrombogenic components to the bloodstream and initiates thrombus formation, the key event underlying ACS [13–15].

The impaired phagocytic capacity of macrophages observed in patients with CHD and ACS may result from several interrelated molecular alterations. One potential mechanism involves the proteolytic cleavage of Mer tyrosine kinase (MerTK), a receptor critical for efferocytosis. Inflammatory activation and increased metalloproteinase activity can lead to the shedding of membrane-bound MerTK, generating a soluble form that disrupts apoptotic cell clearance and contributes to necrotic core expansion within atherosclerotic plaques [16]. In addition, dysregulation of scavenger receptors such as CD36 and SR-A1 alters lipid uptake and receptor signaling, promoting excessive accumulation of oxidized lipids and transformation into foam cells [17]. This lipid overload can perturb membrane fluidity and receptor trafficking, thereby further impairing phagocytic efficiency. Moreover, intracellular lipid accumulation induces lysosomal and endosomal dysfunction, resulting in defective degradation of engulfed material and heightened cellular stress or cytotoxicity [18]. Collectively, these mechanisms—MerTK cleavage-mediated efferocytosis failure, scavenger receptor dysregulation, and lipid-induced lysosomal impairment—provide a plausible explanation for the reduced macrophage phagocytic function observed in patients with CHD and ACS.

Previous studies have substantially clarified the pathophysiological roles of macrophages in the arterial wall. However, the molecular mechanisms regulating MDMs remain less defined. Increased circulating leukocyte counts have been consistently associated with elevated ASCVD risk, with this association largely attributed to the proatherogenic functions of monocytes and neutrophils [19, 20]. Elevated monocyte counts serve as an independent predictor of ASCVD, with monocytosis causally linked to both accelerated progression of atherosclerotic plaques and impaired plaque regression [21, 22]. In line with previous studies, this research found that patients with CHD exhibited higher levels of peripheral blood leukocytes, neutrophils, and monocytes, which were primarily driven by the elevated counts in those with ACS. Therefore, monocytes derived from the peripheral blood of study subjects were induced to differentiate into macrophages, after which their phagocytic activities were systematically assessed. Findings revealed that macrophages in CHD patients, especially those with ACS, showed impaired phagocytic capacity. In future studies, mechanistic analyses will be performed to investigate the link between the functional characteristics of peripheral blood MDMs and the pathogenesis of AS.

This study has several limitations. First, the study was limited by its single-center design and relatively small sample size, and the moderate AUC values for CHD (0.679) and ACS (0.669) indicate only modest discriminative ability, suggesting that the predictive performance should be interpreted with caution and further validated or optimized in larger, multiethnic, multicenter cohorts to improve robustness and generalizability. Second, we only collected blood samples from patients with ACS upon admission and assessed their macrophage phagocytosis. Previous studies have demonstrated that inflammatory markers rise rapidly during the acute phase of ACS. Thus, we hypothesize that macrophage phagocytosis in ACS patients also undergoes rapid changes. Due to ethical limitations, repeated blood sampling was not feasible. Future research will further explore the temporal dynamics of macrophage phagocytosis from the onset of ACS across multiple time points, aiming to elucidate the mechanisms underlying AS and ACS. Third, the current study was unable to delineate the molecular mechanisms connecting macrophage phagocytosis in the blood to AS. In the pathogenesis of AS, M1 macrophages promote inflammation and plaque instability, whereas M2 macrophages suppress inflammation and facilitate tissue repair and plaque stabilization. Therefore, this study focused on M1 macrophages. Future studies comparing the phagocytic profiles of M1 and M2 macrophages are warranted to better reflect the diversity of the plaque microenvironment.

## 5. Conclusion

In summary, the present study indicates that macrophage phagocytic rates are reduced in CHD patients compared to controls, particularly in those with ACS. Reduced macrophage phagocytic activity was identified as an independent predictor of both CHD and ACS. Macrophage phagocytosis may act as a protective biomarker in CHD and ACS, providing new insights into the molecular mechanisms of AS. Additional large-scale clinical investigations and basic research are needed to elucidate the association between macrophage phagocytosis and AS.

## Figures and Tables

**Table 1:** Baseline clinical parameters of controls and CHD.

**Variables**	**Control (** **n** = 270**)**	**CHD (** **n** = 270**)**	**p** ** value**
Age (years)	62.1 ± 11.0	62.3 ± 12.0	0.872
Male, *n* (%)	180 (66.7)	180 (66.7)	1.000
BMI (kg/m^2^)	24.6 ± 3.9	24.9 ± 3.7	0.417
Medical history			
Hypertension, *n* (%)	98 (36.3)	115 (42.6)	0.159
T2DM, *n* (%)	60 (22.2)	79 (29.3)	0.076
Family history of CHD, *n* (%)	36 (13.3)	43 (15.9)	0.465
Smoking history, *n* (%)	73 (27.0)	97 (35.9)	0.033
Drinking history, *n* (%)	67 (24.8)	60 (22.2)	0.543
Medications, *n* (%)			
ACEI/ARB/ARNI	45 (16.7)	46 (17.0)	0.999
*β*-blocker	28 (10.4)	36 (13.3)	0.351
CCB	68 (25.2)	80 (29.6)	0.289
Metformin	44 (16.3)	52 (19.3)	0.431
Diagnostic tests			
SBP (mmHg)	127.9 ± 13.5	130.0 ± 15.2	0.102
DBP (mmHg)	71.5 ± 8.3	72.5 ± 9.6	0.173
Leukocyte count (10^9^/L)	6.9 ± 1.2	7.3 ± 1.6	0.001
Neutrophil count (10^9^/L)	4.2 ± 1.2	4.6 ± 1.5	0.002
Lymphocyte count (10^9^/L)	2.1 ± 0.6	2.1 ± 0.6	0.845
Monocyte count (10^9^/L)	0.35 ± 0.14	0.43 ± 0.20	< 0.001
Total protein (g/L)	73.1 ± 4.2	73.6 ± 5.2	0.281
Globulin (g/L)	30.0 ± 3.1	30.0 ± 3.2	0.892
ALT (U/L)	21.8 ± 7.6	21.8 ± 8.4	0.927
Total bilirubin (*μ*mol/L)	12.0 ± 2.5	11.9 ± 3.2	0.888
TG (mmol/L)	1.6 ± 0.8	1.7 ± 0.9	0.437
TC (mmol/L)	5.3 ± 1.1	5.7 ± 1.6	< 0.001
HDL-C (mmol/L)	1.1 ± 0.2	1.1 ± 0.3	0.075
LDL-C (mmol/L)	3.4 ± 1.1	3.9 ± 1.3	< 0.001
FPG (mmol/L)	5.2 ± 1.0	5.3 ± 1.3	0.294
HbA1c (%)	6.0 ± 0.9	6.1 ± 1.0	0.610
SCr (mmol/L)	95.3 ± 24.6	98.9 ± 30.6	0.132
Urea (mmol/L)	5.3 ± 2.3	5.6 ± 1.1	0.102
Uric acid (mmol/L)	321.8 ± 76.4	326.0 ± 79.0	0.526
hs-CRP (mg/L)	2.8 ± 1.6	4.2 ± 3.5	< 0.001
Fibrinogen (g/L)	3.0 ± 0.6	3.8 ± 1.6	< 0.001
LVEF (%)	62.7 ± 7.7	60.8 ± 9.1	0.009

*Note:* Data are presented as mean ± SD, or *n* (%).

Abbreviations: ACEI, angiotensin-converting enzyme inhibitor; ALT, alanine aminotransferase; ARB, angiotensin receptor blocker; ARNI, angiotensin receptor-neprilysin inhibitor; BMI, body mass index; CCB, calcium channel blocker; CHD, coronary heart disease; DBP, diastolic blood pressure; FPG, fasting blood glucose; HbA1c, glycated hemoglobin A1C; HDL-C, high-density lipoprotein cholesterol; hs-CRP, high-sensitivity C-reactive protein; LDL-C, low-density lipoprotein cholesterol; LVEF, left ventricular ejection fraction; SBP, systolic blood pressure; SCr, serum creatinine; T2DM, Type 2 diabetes mellitus; TC, total cholesterol; TG, triglyceride.

**Table 2:** Baseline clinical parameters and CAG findings of SAP and ACS.

**Variables**	**SAP (** **n** = 146**)**	**ACS (** **n** = 124**)**	**p** ** value**
Age (years)	64.5 ± 10.4	59.7 ± 13.1	< 0.001
Male, *n* (%)	96 (65.8)	84 (67.7)	0.796
BMI (kg/m^2^)	24.9 ± 3.3	24.9 ± 4.0	0.919
Medical history			
Hypertension, *n* (%)	64 (43.8)	51 (41.1)	0.711
T2DM, *n* (%)	43 (29.5)	36 (29.0)	0.999
Family history of CHD, *n* (%)	27 (18.5)	16 (12.9)	0.244
Smoking history, *n* (%)	51 (34.9)	46 (37.1)	0.799
Drinking history, *n* (%)	34 (23.3)	26 (21.0)	0.663
Medications, *n* (%)			
ACEI/ARB/ARNI	25 (17.1)	21 (16.9)	0.999
*β*-blocker	19 (13.0)	17 (13.7)	0.999
CCB	48 (32.9)	32 (25.8)	0.230
Metformin	35 (24.0)	17 (13.7)	0.044
Diagnostic tests			
SBP (mmHg)	130.4 ± 12.1	129.4 ± 18.3	0.588
DBP (mmHg)	72.9 ± 8.4	72.1 ± 10.9	0.480
Leukocyte count (10^9^/L)	6.9 ± 1.3	7.8 ± 1.7	< 0.001
Neutrophil count (10^9^/L)	4.2 ± 1.3	5.0 ± 1.6	< 0.001
Lymphocyte count (10^9^/L)	2.1 ± 0.6	2.1 ± 0.5	0.407
Monocyte count (10^9^/L)	0.4 ± 0.1	0.5 ± 0.2	< 0.001
Total protein (g/L)	73.2 ± 5.4	74.0 ± 5.0	0.247
Globulin (g/L)	30.1 ± 3.6	29.8 ± 2.7	0.379
ALT (U/L)	22.2 ± 8.6	21.4 ± 8.1	0.422
Total bilirubin (*μ*mol/L)	12.1 ± 3.3	11.8 ± 3.0	0.545
TG (mmol/L)	1.7 ± 0.9	1.7 ± 1.0	0.993
TC (mmol/L)	5.8 ± 1.5	5.7 ± 1.6	0.668
HDL-C (mmol/L)	1.1 ± 0.3	1.1 ± 0.3	0.582
LDL-C (mmol/L)	3.9 ± 1.2	3.9 ± 1.5	0.871
FPG (mmol/L)	5.2 ± 1.1	5.4 ± 1.6	0.312
HbA1c (%)	6.1 ± 1.1	6.1 ± 1.0	0.608
SCr (mmol/L)	99.5 ± 32.4	98.2 ± 28.4	0.735
Urea (mmol/L)	5.6 ± 1.1	5.5 ± 1.1	0.278
Uric acid (mmol/L)	326.6 ± 80.2	325.4 ± 78.1	0.897
hs-CRP (mg/L)	2.8 ± 1.8	5.8 ± 4.2	< 0.001
Fibrinogen (g/L)	3.1 ± 0.8	4.8 ± 1.8	< 0.001
LVEF (%)	61.6 ± 8.0	59.8 ± 10.1	0.099
Number of involved vessels, *n* (%)			< 0.001
Single vessel	57 (39.0)	25 (20.2)	
Double vessel	69 (47.3)	62 (50.0)	
Triple vessel	20 (13.7)	37 (29.8)	
Type of involved vessels, *n* (%)			
Left main	12 (8.2)	13 (10.5)	0.536
LAD	96 (65.8)	99 (79.8)	0.014
LCX	68 (46.6)	67 (54.0)	0.272
RCA	91 (62.3)	94 (75.8)	0.019

*Note:* Data are presented as mean ± SD, or *n* (%).Other abbreviations were as in Table 1.

Abbreviations: ACSs, acute coronary syndromes; CAG, coronary angiography; LAD, left anterior descending artery; LCX, left circumflex artery; RCA, right coronary artery; SAP, stable angina pectoris.

**Table 3:** Multivariable linear regression analysis of macrophage phagocytosis.

**Variables**	**β**	**95% CI**	**p** ** value**
Age	−0.36	−0.39 to −0.32	< 0.001
LDL-C	−0.85	−1.64 to −0.07	0.03
hs-CRP	−0.16	−0.31 to −0.02	0.03
Fibrinogen	−0.84	−1.18 to −0.50	< 0.001

*Note:* Adjusted for age, BMI, SBP, DBP, total protein, globulin, total bilirubin, ALT, TG, TC, HDL-C, LDL-C, FPG, HbA1c, urea, SCr, uric acid, leukocyte, neutrophil, lymphocyte, monocyte, hs-CRP, fibrinogen, and LVEF. Other abbreviations were as in Table 1.

Abbreviations: *β*, multiple regression coefficient; CI, confidence interval.

**Table 4:** Logistic regression analysis for the presence of CHD and ACS.

	**Adjustment**	**OR**	**95% CI**	**p** ** value**
CHD	Model 1	0.89	0.86 to 0.92	< 0.001
	Model 2	0.81	0.78 to 0.85	< 0.001
	Model 3	0.85	0.80 to 0.89	< 0.001

ACS	Model 1	0.89	0.86 to 0.93	< 0.001
	Model 2	0.75	0.70 to 0.80	< 0.001
	Model 3	0.75	0.68 to 0.82	< 0.001

*Note:* Model 1: unadjusted. Model 2: adjusted for age and sex. Model 3: adjusted for age, sex, BMI, SBP, DBP, total protein, globulin, ALT, total bilirubin, TG, TC, HDL-C, LDL-C, FPG, HbA1c, urea, SCr, uric acid, leukocyte, neutrophil, lymphocyte, monocyte, hs-CRP, fibrinogen, LVEF, hypertension, T2DM, smoking history, drinking history, and family history of CHD. Other abbreviations were as in Table 1.

Abbreviations: CI, confidence interval; OR, odds ratio.

## Data Availability

The data are available from the corresponding author upon reasonable request.

## Nomenclature

CHD: coronary heart disease
ASCVD: atherosclerotic cardiovascular disease
AS: atherosclerosis
LDL-C: low-density lipoprotein cholesterol
ox-LDL: oxidized LDL
ACSs: acute coronary syndromes
MDMs: monocyte-derived macrophages
SAP: stable angina pectoris
CAG: coronary angiography
T2DM: Type 2 diabetes mellitus
BMI: body mass index
ALT: alanine aminotransferase
TG: triglyceride
TC: total cholesterol
HDL-C: high-density lipoprotein cholesterol
FBG: fasting blood glucose
HbA1c: glycated hemoglobin A1c
SCr: serum creatinine
hs-CRP: high-sensitivity C-reactive protein
RCA: right coronary artery
LAD: left anterior descending artery
LCX: left circumflex artery
PBMCs: peripheral blood mononuclear cells
LPS: lipopolysaccharide
IFN*γ*: interferon-*γ*
SD: standard deviation
ANOVA: analysis of variance
LSD: least significant difference
ROC: receiver operating characteristic
AUC: area under the curve
SBP: systolic blood pressure
DBP: diastolic blood pressure
TNF-*α*: tumor necrosis factor-*α*
IL-1*β*: interleukin-1*β*
MerTK: Mer tyrosine kinase
